# The Rubber Patents

**Published:** 1868-12

**Authors:** 


*The Rubber Patents.*—Although the Goodyear Company has succeeded in obtaining injunctions against the Cincinnati Dentists, who have, for many months past, been manfully resisting their attempt to force them to obtain licenses, and the "Simpson Rubber" has been declared an infringement on the Goodyear patent, yet this Company has been unsuccessful in their suit against Dr. Jno. B. Newbrough the originator of the "Iodized Rubber," as this compound has been declared, by the U. S. Court for the Southern District of New York, to be no infringment of the Goodyear patent.

The Goodyear Company claimed that the hardening of the "Iodized Rubber" was due to the sulphur it contained (two-thirds of an ounce to the pound,) and was therefore an infringement.

The testimony of Profs. Silliman, Chandler, Seely and others, was presented at the trial to establish the following facts: 1. That the sulphur had no influence in hardening iodized rubber, and was only used for the purpose of neutralizing the effects of the iodine upon the vermilion, which was used to color the rubber. 2. That iodized rubber would not harden in steam. 3. That iodized rubber hardened equally well without any sulphur in it at all. 4. That the iodized and vulcanized rubbers when hardened were chemically two different substances.

As this iodized rubber is highly recommended by many who have used it, the following method may prove interesting to those who may wish to do so: "The plaster model is made of plaster and white sand, in the proportion of two parts of plaster to one of sand, mixed with water, to which a little salt has been added. The process is the same as for the Goodyear rubber, till the case is ready for the flask. In investing the case, use plaster and sand same as for the model. Warm slightly, separate the flask and remove the wax plate and all the wax from about the teeth. Cut outlets for surplus rubber, coat the surface of the moulds with liquid silex, close the flask and place it in the oven. Apply heat, and let the flask remain subjected to a moderate degree of heat for eight or twelve hours; the object being to thoroughly dry the moulds. The drying of the moulds must not be neglected in working this rubber, as it will not harden over damp plaster. When thoroughly dry, pack while hot, being careful not to have it too hot. More difficulty is experienced in packing this rubber than in that of the Goodyear Company, hence more care is necessary in closing the flask, to avoid breaking the teeth. To vulcanize, heat up gradually, to 380° in one hour, keep it at that heat for forty minutes. Cool perfectly before attempting to open the flask. The iodized rubber plate is finished as plates made from the other compounds are."

